# Microplastic Contamination of Wild and Captive Flathead Grey Mullet (*Mugil cephalus*)

**DOI:** 10.3390/ijerph15040597

**Published:** 2018-03-26

**Authors:** Lewis T. O. Cheung, Ching Yee Lui, Lincoln Fok

**Affiliations:** 1 Department of Social Sciences, The Education University of Hong Kong, Tai Po, New Territories, Hong Kong, China; ltocheung@eduhk.hk (L.T.O.C.); luicy@eduhk.hk (C.Y.L.); 2 Department of Science and Environmental Studies, The Education University of Hong Kong, Tai Po, New Territories, Hong Kong, China

**Keywords:** microplastic, ingestion, captive mullets, wild mullets, marine pollution

## Abstract

A total of 60 flathead grey mullets were examined for microplastic ingestion. Thirty wild mullets were captured from the eastern coast of Hong Kong and 30 captive mullets were obtained from fish farms. Microplastic ingestion was detected in 60% of the wild mullets, with an average of 4.3 plastic items per mullet, while only 16.7% of captive mullets were found to have ingested microplastics, with an average of 0.2 items per mullet. The results suggested that wild mullets have a higher risk of microplastic ingestion than their captive counterparts. The most common plastic items were fibres that were green in colour and small in size (<2 mm). Polypropylene was the most common polymer (42%), followed by polyethylene (25%). In addition, the abundance of microplastics was positively correlated with larger body size among the mullets.

## 1. Introduction

Plastic constitutes the largest portion of marine litter in the world, it was estimated that up to 12.7 million tons of plastic debris enter the oceans every year [1]. Plastic debris has been found on the surface of open oceans from the poles to the equator [2,3,4,5], along shorelines, as well as in deep-sea sediments [6,7,8]. The larger plastic items that have been found in the ocean pose threats, including suffocation, entanglement, and ingestion, to wildlife such as birds, sea turtles, marine mammals, invertebrates, and fish. The potential biological consequences to wildlife include damage to moving, feeding and reproduction ability, ulcerations, and even death [9,10,11,12].

Microplastics are plastic debris with diameters less than 5 mm [13], and the major sources of microplastics can be categorised into primary and secondary sources. Primary microplastics refer to particles originally manufactured in a particular size for specific applications. They include pellets that were used to manufacture plastic products and microbeads that are used in cosmetic, personal cleansing, and household products [14]. These microplastics cannot be completely removed by wastewater plants and end up in the oceans [9,15,16]. The breakdown of larger plastic debris by photochemical, mechanical, and biological processes in the marine environment results in secondary microplastics [10]. As most plastics were designed to be non-biodegradable, they only break down into smaller sizes and exist in the natural environment for hundreds or even thousands of years [3]. At the same time, the wastewater from washing clothes also discharges synthetic fibres to the marine environment [17,18].

Previous research has suggested that the sizes of microplastics that are equivalent to the sizes of plankton, and thus have a high potential to be mistaken as food, are ingested by a wide range of marine species [19]. Evidence has shown that ingested microplastics may remain in the digestive tract, causing injury and internal blockages that hinder food digestion processes [10,20]. Microplastic ingestion may also pose risks of degradation in feeding, respiratory, and reproductive functions and could change molecular and cellular pathways [11,21]. Moreover, the accumulation of plastics in the digestive tract may provide a false signal of being full; thus, the organism may reduce its food consumption [22]. In addition, liver toxicity [23] and vertical migration due to increased buoyancy have been found to be potential effects of plastic ingestion on marine fish [24]. A recent study also suggested that environmentally relevant concentrations of microplastics in juvenile *Perca fluviatilis* (European perch) may affect their rates of survival [25].

There are further concerns about organic contaminants adsorbed by, or exist within microplastics themselves can be transferred to marine species and enter the food web. Toxicological risks may then transfer to higher trophic levels [19,26]. Microplastics are believed to carry more pollutants than larger plastics, as the former are usually ingested by organisms at lower trophic levels, and consequently, the potentials for bioaccumulation and biomagnification through the food chain are higher [18]. Although the health risks of microplastics to the human body remain to be unknown, there has been general concern that the potential risks on wildlife that are posed by microplastics may eventually transfer to humans, who are apex predators [27,28]. Microplastic pollution can thereby also be a food safety issue, especially in countries where fish is a staple food [27,28,29].

Approximately 150 fish species worldwide have been reported to ingest microplastics [30], including but not limited to, samples from the Northwest Atlantic [31], the English Channel [19], the Mediterranean Sea [32], Australia [29], the Adriatic Sea [33], and China [30]. The ingestion of microplastics by fish is associated with the abundance of microplastic pollution in the local marine environments and the feeding methods of the fishes [32,34]. Only a limited number of studies have documented microplastic ingestion by fish in Hong Kong, even though the city was determined to be a hotspot of microplastic pollution, with mean microplastic abundance values of 5595 items/m^2^ along the shorelines [35]. Fok and Cheung [35] suggested that this level is above the international average, which indicated the high potential risk of microplastic ingestion in this area. In addition, the study suggested that the direct inputs of microplastics were from human activities, such as spillage and littering from marine vessels; large plastic debris from coastal areas is also a major source of microplastics in Hong Kong. Thus, it is essential to investigate the ingestion of microplastic by fishes in Hong Kong, which is a coastal city with more than 20 fish culture zones that provide seafood for local consumption and marine parks that conserve the marine resources with high ecological value. Moreover, there are three different commercial fish-feeding environments in Hong Kong, including fish culture zones, the open sea, and aquaculture farms, which may result in differences in the extent of microplastic ingestion due to different feeding methods and the level of microplastic pollution in the feeding environments.

The flathead grey mullet (*Mugil cephalus*) is an omnivorous fish that possess a high risk for microplastic ingestion [36]. As an economically important species that contributes to fisheries in many countries, *M. cephalus* constitutes 2.6% of the total marine fish production [37]. Mullet are demersal fish, and its benthic feeding habits increase the risk of microplastic ingestion from contaminated sediments [36]. In addition, mullet are considered to be an important indicator species and a biomarker for monitoring the health of coastal ecosystems worldwide [36]. Moreover, grey mullet have been found to be a species that ingests microplastics [30,38], and is potentially related to microplastic transfer from lower to higher trophic levels as it is consumed by birds and carnivorous fishes [39]. This species also has commercial importance and is commonly available in the Hong Kong market; thus, it was selected for investigation in this research. As plastic items that were detected in this research were found in seafood consumed by humans, the results of this study is relevance to food safety and public health concerns [40].

The primary goal of this paper is to determine the extent of microplastic ingestion by mullets in Hong Kong. More specifically, the differences in terms of abundance of microplastics between wild and captive grey mullet are measured. Secondly, the relationships between the abundance of microplastics and the physical characteristics of mullet samples are investigated. To achieve these study objectives, ingestion of micro- and mesoplastics was investigated in wild and captive grey mullet. The abundance, type, size, colour, and composition of microplastics obtained from the stomachs and intestines of mullet were recorded for investigation and analysis.

## 2. Materials and Methods

### 2.1. Sample Collection

From February to March 2017, 60 flathead grey mullet (*Mugil cephalus*) samples were purchased from the fresh markets in Hong Kong. Among the samples, 30 wild mullets were collected from the eastern coast near Sam Mun Tsai, while another 30 captive mullets were collected from local fish ponds. The total weight and fork length of each sample were measured to the nearest 0.1 g and 0.1 cm, respectively [19,30,32]. The stomachs and intestines were removed by ventrally dissecting the sample, and the weights (to the nearest 0.1 g) of the stomachs and intestines were measured by using an electronic weight balance. Then, tissue samples were kept in a −20 °C refrigerator before the following procedures.

### 2.2. Experiment Process and Procedure

Hydrogen peroxide (H_2_O_2_) digestion was carried out based on the protocol that was described by Jabeen et al. [30]. The extracted stomach and intestine portions were placed into clean one-litre beakers and digested to separate the plastic from the tissue. Approximately 400 mL of 30% hydrogen peroxide was added to digest the organic matter, and the beakers were covered with clean ceramic tiles and placed on a hotplate stirrer at approximately 65 °C for 24 to 72 h. The dissolved solution was retained upon completion of the digestion.

The filtration and density separation of plastic particles by saline (NaCl) solution, followed the methods suggested by Jabeen et al. [30]. Approximately 800 mL of saturated NaCl solution (1.2 g/mL) was added to the beaker for floatation by increasing the density of the solution. The solution was stirred and mixed, and then kept overnight, which allowed for the separation and floatation of plastics debris from the dissolved tissue solution due to the density differences. Next, the solution was vacuum filtered through Whatman Grade 1 filter paper (11 µm pore size, Whatman plc, Maidstone, UK). The filters were stored in clean Petri dishes that were covered with lids after filtration and prepared for microscopic observation.

The filters were observed under a stereomicroscope (SZ1145, Olympus, Tokyo, Japan), and plastic particles were observed, visually identified, and captured by a camera for the records. The types of plastic items were categorised based on the physical characteristics described by Li et al. [41], including fibres, which showed elongated and slender shapes; fragments, which appeared as incomplete debris of larger plastic; and sheets, which are thin layers of plastic. The longest dimensions (measured in mm) and the colours of the identified plastic items were also recorded. Moreover, the identified plastic items were assigned to the size classes that were used by Romeo et al. [42]; items smaller than 2 mm were listed as small microplastics, while items between 2 and 5 mm were listed as large microplastics, and those with sizes larger than 5 mm were considered to be mesoplastics.

Identified plastic items with different physical characteristics were randomly sampled and tested by a Fourier transform infrared (FT-IR) spectrometer (PerkinElmer Frontier, Schwerzenbach, Switzerland) equipped with a universal attenuated total reflectance (UATR) sampling accessory with a spectral range of 4000–675 cm^−1^ and 16 co-scans for every measurement.

All of the equipment, including the glassware and dissection apparatus were rinsed three times with deionised water to prevent contamination [30,41,43]. Saline solution and hydrogen peroxide were filtered with Whatman Grade 1 filter paper (11 µm pore size) prior to use. Laboratory coats and gloves were worn during the experiment process. Additionally, samples were immediately covered when not in use. Filter paper soaked with deionised water was placed in the laboratory environment to collect the background air-borne contamination. No plastic items were detected from the background air.

### 2.3. Statistical Analysis

The collected data were analysed by SPSS 21 (IBM, New York, NY, USA). Because plastic abundance data in this study do not approach a normal distribution, an independent-samples Mann-Whitney U test was used to demonstrate the differences in plastic abundances between wild mullet and captive mullet in terms of number of ingested plastic and average plastic item size found per individual. Because data distributions between the two sample groups are different, the mean along with the median values are reported in this study. The former measure of central tendency is also more widely adopted by similar studies. In addition, a correlation analysis was performed to examine the relationship between the abundance of plastic and the physical characteristics of the sampled fish. The microplastic abundance was presented in terms of average size of each item and number of items per individual mullet, while the physical characteristics of the mullet, included the body weight, fork length, and total weight of the intestine and stomach. A 0.05 level of significance was adopted.

## 3. Results and Discussion

### 3.1. Abundance of Plastic in Mullet

The physical characteristics of both wild and captive mullets are shown in Table 1. Plastic items were found in both the wild and captive mullet samples. A total of 129 plastic items were found in 18 wild fish samples, which suggested that 60% of the wild fish samples contained plastic items. The occurrence of microplastic ingestion found in this study approaches the upper bound among similar studies of fishes [44]. Moreover, only six plastic items were found from five out of the 30 captive samples (16.7%). The mean number of plastic items found in the wild mullet samples is 4.3 items per mullet, which is higher than the 3.7 and 3.8 items per mullet that were observed in China [30] and South Africa [38], respectively. High densities of plastics are known to exist in these coastal areas, with mean densities of 3.97 and 7.03 items per m^3^ having been observed in estuaries of South China and South Africa, respectively [5,45].

**Figure 1:**
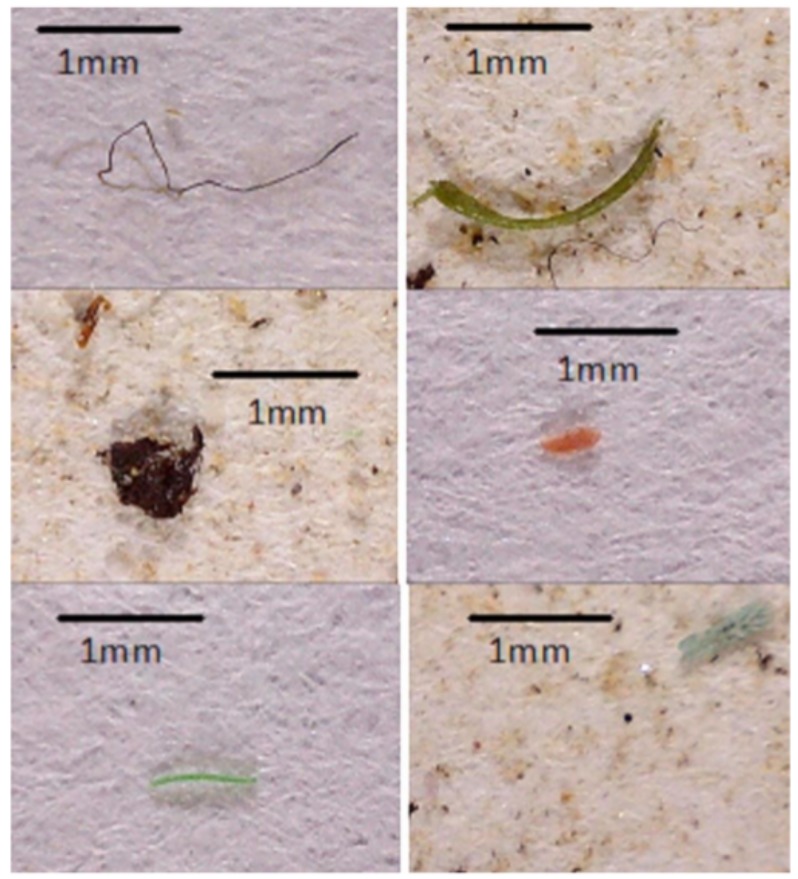
Microplastics found in the stomachs and intestines of mullet.

The mean number of plastic items was significantly higher in wild mullets than in captive mullets (U = 233; *p* = 0.000). On average, 4.3 plastic items were found in wild mullets, while only 0.2 plastic items were observed in their captive counterparts. In contrast, no significant difference was detected for the average sizes of ingested plastics between wild and captive mullets (U = 44; *p* = 0.941). An average size of 1.18 ± 0.77 (SD) mm was observed for the ingested plastic in total mullets. Micrograms of selected microplastic pieces are shown in Figure 1.

The higher abundance of microplastics in wild mullets may be related to the differences in the habitats and the occurrence of plastic items in the feeding environment [46]. Wild mullets live in the open sea where microplastics are abundant. According to Tsang et al. [18], microplastics in the marine environment of Hong Kong are mainly introduced by local land-based sources, including sewage discharge, illegal dumping, stormwater runoff, industrial activities, and accidental spillage during transportation. Plastic waste from the coastal and beach areas may break down into smaller microplastics in the marine environment [10,35]; daily municipal and industrial sewage discharge may also transport microbeads and synthetic fibres into the sea [17,18]. In addition, the open sea near Hong Kong also receives urban runoff from densely populated urban regions that consist of residential, industrial, and commercial areas. The runoff is especially pronounced during the wet season when extensive amounts of plastic debris may be washed into the sea. In contrast, the seawater used in fish ponds is pumped into the ponds by circulation pumps, and the inlets of these pumps are located well beneath the sea surface where the majority of floating plastic items exist. In addition, the primary screening of the pump system also prevents larger plastic items from entering the ponds. These conditions explain a lower risk of microplastic ingestion in fish pond environments.

Although there is still uncertainty about the safety of microplastic ingestion by humans, this research provided evidence that microplastics exist in mullet that are consumed by Hong Kong residents. Although gastrointestinal tracts are usually removed before human consumption, ingestion of microplastics by table fishes, such as mullet, can involve potential health risks for consumers due to the bioaccumulation of marine pollutants, as mentioned above. The results from this study suggested that captive mullets might pose a lower risk to human health than do wild mullets. Thus, captive mullets are a less risky choice for human consumption in this regard. Nevertheless, it should also be noted that such a health risk has not been quantified by the scientific community.

Moreover, the number of plastic items found in the mullet samples was highly varied (from 0 to 80 items). The accumulation of plastic may depend on the size of the gastrointestinal tract and the ingested plastic items. Some plastic items may be small enough to be expelled from the fish through the faeces, and inversely, some comparatively larger items may be trapped in the body tissues [47,48]. Uncertainty remains about the residence time of plastics that accumulate in the digestive system as well as the potential translocation of plastic items in mullets [19]; thus, further research is required to enhance the reliability of the data.

### 3.2. Types, Colours and Sizes of Plastic Items in Mullet

Fibres were the most common type of plastic found in the mullet samples and were the only type of plastic obtained from captive mullet. At the same time, more types of plastic items were found in wild mullet. A total of 77 plastic fibres were observed in wild mullet, which constituted approximately 60% of the total number of plastic items that are found in wild mullet. A total of 44 plastic fragment items, which constituted 34% of the total, were found in wild mullet; and, the remaining 6% was identified as plastic sheets (Table 2). Fibres were also found to be the most common microplastic type in previous studies [19,27,30,44]. In addition, these findings are also similar to those from previous studies on microplastic ingestion in wild mullet [30,38] that identified fibre as the major type of microplastic, followed by fragments. Previous research has also revealed that more fibres were found in benthic species, which is likely linked to the relatively high abundance of fibres on the seabed when compared to other habitats [27,46]. In addition, mullet are a polychaete-preying species; it is possible for them to mistake fibres for polychaetes [49].

Eight colours of plastic items were observed in the wild mullets, and there were a higher variety of colours in wild mullets than captive mullets, in which only four colours were found. The majority of plastic items found in wild mullet were green, constituting approximately 44% of the total number of items, followed by blue (16%) and black (15%). Other plastic items that were red, colourless, white, purple, and orange were also observed (Table 2). Plastic items with dark colours were commonly found in the digestive systems of mullet, implying that fishes may mistakenly consume these plastic items as food as the sizes and colours of these plastic items were similar to those of plankton in the marine environment [19].

Microplastics that were smaller than 5 mm accounted for 96% and 100% of the total number of plastic items in wild mullets and captive mullets, respectively. The sizes of microplastics and mesoplastics that were identified ranged from 0.1 to 4.9 mm and 5.5 to 12 mm, respectively, in the wild mullet samples. At the same time, the majority of microplastics that were found in both samples were smaller than 2 mm, which constituted 90% and 83% of the total number of items, respectively (Table 2). These results were similar to those recorded in Jabeen et al. [30], who found that small microplastic items less than 2 mm were dominant, followed by large microplastics (2–5 mm) and mesoplastics (>5 mm).

### 3.3. Identification and Validation of Plastic Items

Out of 140 visually identified plastic items, 84 items (60%) were sampled for FT-IR identification. A total of nine polymer types were identified, and 6% of the visually identified items were determined to be non-plastic by FT-IR spectroscopy, which revealed a success rate of 94% in this research. Of the identified plastic polymer types, polypropylene contributed the majority (42%), followed by polyethylene (25%) and polyester (16%) (Table 2). In addition, other polymer types, including polyethylene terephthalate (6%), nylon (4%), and polytetrafluoroethylene (3%) were also identified. A similar result was observed by Tsang et al. [18], who found that polypropylene was the major polymer (50.9%), followed by polyethylene (44.6%) in coastal water and sediment samples collected from the Hong Kong marine environment. Tsang et al. [18] claimed that this result was not surprising, as polypropylene and polyethylene constituted more than 60% of plastic production. The possible sources of polypropylene include the widespread usage of polypropylene rope, which is used for attachment and in predator exclusion netting [41]. Polyethylene is common in plastic packaging materials, such as plastic bags and sheets. This type of terrestrial plastic litter may easily enter the natural marine environment through littering in coastal areas or may even be blown by the wind due to its low-density characteristics [50].

### 3.4. Abundance of Microplastics and Physical Characteristics of Mullet

The data collected were analysed by Spearman’s rho test to explore the correlations between the variables (abundance of microplastics and physical characteristics of mullet). The abundance of microplastics was quantified by both the number and the mean size of ingested plastic items, while the physical characteristics of the mullet included the body weight, fork length, as well as the intestine and stomach weights. The wild mullet, captive mullet, and total mullet were tested.

The results from the tests on wild mullet and total mullet suggested positive correlations between the abundance of microplastics and the physical characteristics of mullet, including intestine weight, fork length, and body weight (Table 3). As a higher number of ingested microplastics was observed when the stomach of mullet was full, the presence of microplastic particles in fishes could be a temporary phenomenon.

## 4. Conclusions

The results of this study indicated that wild mullets are more likely to ingest microplastics than captive mullets. In addition, this study also suggested correlations between the abundance of microplastics and the physical characteristics of mullet samples, which provided evidence of microplastics ingestion and demonstrated the significant impacts on the physical characteristics of mullets. International government and academic efforts are needed to establish universal protocols to further investigate microplastics in biota and assess the risks of microplastic ingestion to wildlife and humans. 

More importantly, actions should be taken at the individual, local, and global level to tackle the problem by implementing source controls that will keep plastic from reaching our seas, which is the most effective precautionary approach to address the issue in the long run. At the individual level, one can reduce the sources by reducing the usage of disposable plastic items, such as plastic bags, boxes, bottles, and straws, and individuals can bring their own reusable items. Some countries, such as the United States, Canada, Australia, and the United Kingdom, have already attempted to ban microbeads in personal care products through legislation [49]. In addition, governments and relevant organisations should seriously consider other measures and incentives to reduce plastic packaging as well as other plastic waste. Moreover, further research is needed to substantially enhance the effectiveness of wastewater plants to reduce the number of microplastics that aare entering the sea.

## Figures and Tables

**Table 1:** Descriptive statistics of wild (Sam Mun Tsai; *n* = 30), captive (Yuen Long; *n* = 30) and total (*n* = 60) mullet samples. M: mean; Mdn: median; SD: standard deviation; IQR: interquartile range.

Group	Wild				Captive				Total			
Statistics	M	Mdn	SD	IQR	M	Mdn	SD	IQR	M	Mdn	SD	IQR
Number of plastic items	4.30	1.00	14.5	2.25	0.20	0.00	0.48	0.00	2.25	0.00	10.4	1.00
Size of plastic items (mm)	1.21	0.975	0.833	1.08	1.05	1.10	0.51	0.875	1.18	1.00	0.767	1.05
Fish length (mm)	433	431	19.2	25.0	397	400	10.4	15.8	415	409	23.7	32.5
Fish weight (g)	765	766	104	104	716	719	44.8	65.4	741	739	82.9	91.7
Tissue Weight (g)	39.4	37.7	9.60	15.2	21.4	20.7	3.87	4.28	30.4	26.4	11.6	17.3

**Table 2:** Types, colours, sizes and polymer identification of plastic items.

Identification Category	Variable	Wild Mullet	Captive Mullet
Types of plastic	Fibre	77 (60%)	6 (100%)
	Fragment	44 (34%)	0
	Sheet	8 (6%)	0
Colours of plastic	Green	56 (43%)	0
	Blue	21 (16%)	2 (33%)
	Black	19 (15%)	1 (17%)
	Red	10 (8%)	1 (17%)
	Colourless	10 (8%)	0
	White	10 (8%)	0
	Purple	2 (2%)	2 (33%)
	Orange	1 (<1%)	0
Sizes of plastic	Small microplastic (<2 mm)	116 (90%)	5 (83%)
	Large microplastic (2–5 mm)	8 (6%)	1 (17%)
	Mesoplastic (>5 mm)	5 (4%)	0
Polymer identification of plastic	Polypropylene	33 (42%)	
	Polyethylene	20 (25%)	
	Polyester	13 (16%)	
	Polyethylene terephthalate	5 (6%)	
	Nylon	3 (4%)	
	Polytetrafluoroethylene	2 (3%)	
	Others	3 (4%)	

**Table 3:** Correlation coefficients between the abundance of microplastics and the physical characteristics of wild, captive, and total mullets, as determined by Spearman’s rho test.

Variables		Abundance of Microplastics		Physical Characteristics of Mullet		
		Number of Items	Average Size of Items	Body Weight	Fork Length	Intestine and Stomach Weight
***Wild Mullets***						
Abundance of microplastics	Number of items	1				
	Average size of items	0.873 **	1			
Physical characteristics of mullet	Body weight	−0.191	−0.123	1		
	Fork length	0.147	0.143	0.756 **	1	
	Intestine and stomach weight	0.415 *	0.442 *	0.365 *	0.648 **	1
***Captive Mullets***						
Abundance of microplastics	Number of items	1				
	Average size of items	0.995 **	1			
Physical characteristics of mullet	Body weight	0.010	0.041	1		
	Fork length	−0.315	−0.283	0.751 **	1	
	Intestine and stomach weight	−0.014	0.001	0.288	0.287	1
***Total Mullets***						
Abundance of microplastics	Number of items	1				
	Average size of items	0.962 **	1			
Physical characteristics of mullet	Body weight	−0.003	0.042	1		
	Fork length	0.362 **	0.332 **	0.663 **	1	
	Intestine and stomach weight	0.503 **	0.475 **	0.430 **	0.837 **	1

* Significant at the 0.05 level (2-tailed); ** significant at the 0.01 level (2-tailed).
